# Low Enzymatic Activity Haplotypes of the Human Catechol-O-Methyltransferase Gene: Enrichment for Marker SNPs

**DOI:** 10.1371/journal.pone.0005237

**Published:** 2009-04-13

**Authors:** Andrea G. Nackley, Svetlana A. Shabalina, Jason E. Lambert, Mathew S. Conrad, Dustin G. Gibson, Alexey N. Spiridonov, Sarah K. Satterfield, Luda Diatchenko

**Affiliations:** 1 Center for Neurosensory Disorders, School of Dentistry, University of North Carolina, Chapel Hill, North Carolina, United States of America; 2 National Center for Biotechnology Information, National Institutes of Health, Bethesda, Maryland, United States of America; 3 Department of Endodontics, School of Dentistry, University of North Carolina, Chapel Hill, North Carolina, United States of America; 4 Department of Mathematics, Massachusetts Institute of Technology, Cambridge, Massachusetts, United States of America; James Cook University, Australia

## Abstract

Catechol-O-methyltransferase (COMT) is an enzyme that plays a key role in the modulation of catechol-dependent functions such as cognition, cardiovascular function, and pain processing. Three common haplotypes of the human *COMT* gene, divergent in two synonymous and one nonsynonymous (*val* ^158^ *met*) position, designated as low (LPS), average (APS), and high pain sensitive (HPS), are associated with experimental pain sensitivity and risk of developing chronic musculoskeletal pain conditions. APS and HPS haplotypes produce significant functional effects, coding for 3- and 20-fold reductions in COMT enzymatic activity, respectively. In the present study, we investigated whether additional minor single nucleotide polymorphisms (SNPs), accruing in 1 to 5% of the population, situated in the *COMT* transcript region contribute to haplotype-dependent enzymatic activity. Computer analysis of *COMT* ESTs showed that one synonymous minor SNP (rs769224) is linked to the APS haplotype and three minor SNPs (two synonymous: rs6267, rs740602 and one nonsynonymous: rs8192488) are linked to the HPS haplotype. Results from *in silico* and *in vitro* experiments revealed that inclusion of allelic variants of these minor SNPs in APS or HPS haplotypes did not modify COMT function at the level of mRNA folding, RNA transcription, protein translation, or enzymatic activity. These data suggest that neutral variants are carried with APS and HPS haplotypes, while the high activity LPS haplotype displays less linked variation. Thus, both minor synonymous and nonsynonymous SNPs in the coding region are markers of functional APS and HPS haplotypes rather than independent contributors to COMT activity.

## Introduction

Catechol-O-methyltransferase (COMT) is a ubiquitously expressed enzyme that maintains basic biologic functions by inactivating a broad range of catechol substrates, including catecholamines (epinephrine, norepinephrine, and dopamine) and catecholestrogens. The human *COMT* gene is located on chromosome 22, band q11.2 where it encodes two distinct proteins, soluble COMT (S-COMT) and membrane-bound COMT (MB-COMT), through the use of alternative promoters and translation initiation sites [1], [2]. S-COMT is predominately expressed in peripheral tissues, while MB-COMT is predominatly expressed in brain. The specific cell and tissue distribution of these isoforms largely overlaps with that of its catechol substrates.

To date, the role of COMT in catechol metabolism has prompted over 600 investigations of its variants in the etiology of numerous disorders. Functional polymorphisms in the *COMT* gene are associated with dopamine and norepinephrine-dependent neuropsychiatric disorders such as schizophrenia [3], [4], bipolar disorder [5], obsessive compulsive disorder [6], [7], anxiety disorders [8]–[10], attention deficit hyperactivity disorder [11], [12], addiction [13], and anorexia nervosa [14], [15] as well as neurodegenerative disorders such as Parkinson's disease [16], [17]. *COMT* polymorphisms have also been associated with the development of disorders such as cardiovascular disease [18], [19] and estrogen-induced hormonal cancers [20], [21], which are characterized by increased levels of catecholamines and their reactive products in peripheral tissues.

More recently, COMT has been implicated in the modulation of persistent pain. Studies show that reduced COMT activity results in increased pain sensitivity and proinflammatory cytokine production in animal models [22], [23]. These results are consistent with clinical studies demonstrating that facial pain patients exhibit lower COMT activity relative to controls [24]. Furthermore, functional polymorphisms in the *COMT* gene resulting in reduced enzyme activity are associated with fibromyalgia [25], [26], temporomandibular disorder (TMD) onset [27], experimental pain sensitivity [27], [28], and altered morphine efficacy in cancer pain treatment [29]. Collectively, results of these investigations demonstrate that low activity variants of *COMT* negatively impact many aspects of physiology and behavior.

Most of these association studies focused on the common single nucleotide polymorphism (SNP) rs4680 located at codon 158. The minor 675A allele produces a valine to methionine substitution, resulting in a less thermostable COMT enzyme that exhibits a 3-fold reduction in activity [30]. Until late, the nonsynonymous *val* ^158^ *met* allele has been generally accepted as the main source of individual variation in COMT activity. However, observed associations between catechol-related disorders and the *met* ^158^ allele are modest and often inconsistent. In the hope of capturing additional functional *COMT* polymorphisms that contribute to disease phenotype, investigators have begun casting larger nets that extend beyond rs4680. Recent reports have demonstrated associations between the minor alleles of rs2097603 located in the *MB-COMT* promoter region, rs737865 located in intron 1, rs6267 located in exon 3, and rs165599 located in the 3′ untranslated region and enzyme amount/activity as well as COMT-dependent phenotypes [31]–[35]. Thus, emerging evidence suggests that there are multiple functional interacting SNPs within the *COMT* gene locus.

Applying this approach to the study of pain disorders, our group identified three major *COMT* haplotypes consisting of three SNPs in the coding region, rs4633 (C/T), rs4818 (C/G), and the commonly studied rs4680 (G/A), that are strongly associated with experimental pain sensitivity and likelihood of developing TMD [27], [36]. On the basis of subjects' pain responsiveness, haplotypes were designated as low (LPS; CGG), average (APS; TCA), or high (HPS; CCG) pain sensitive. Individuals carrying APS/APS or HPS/APS diplotypes were nearly 2.5 times more likely to develop TMD. In a related study, Vargas-Alarcon and colleagues found that the HPS haplotype was associated with fibromyalgia risk and symptom severity [26]. APS and HPS haplotypes associated with pain sensitivity code for functional changes in COMT. In cells expressing S- or MB-COMT, the APS haplotype displays a modest 3-fold reduction in enzymatic activity likely due to the previously reported decrease in protein thermostability coded by the *met* ^158^ allele. The HPS haplotype, however, exhibits a marked 20-fold reduction in enzymatic activity paralleled by reduced protein translation efficiency due to formation of a longer more stable mRNA secondary structure with a 17 kcal/mol reduction in Gibbs free energy [37]. Thus, combinations of commonly observed alleles in the coding region of the human *COMT* gene can impair the activity of enzyme crucial for a variety of essential functions such as cognition, cardiovascular tone, and pain processing.

While LPS, APS, and HPS haplotypes of the *COMT* gene are common in the population, accounting for nearly 96% of all detected haplotypes in the coding region [27], a significant number of less common minor SNPs are also situated in this region. The presence of such minor SNPs in the major *COMT* haplotypes could be 1) *compensatory*, rescuing COMT activity by annulling the effects of their parent haplotypes [38], 2) *neutral*, carried with common selectively favored variants [39], or 3) *deleterious*, further reducing COMT activity through independent mechanisms or through interactions with other functional variants [40].

Thus, the purpose of the present study was to identify and characterize the potential functional effects of additional SNPs within the *COMT* gene. An EST database search was first performed to identify minor SNPs situated in the transcript region of the *COMT* gene that were linked with the major haplotypes. We identified a total of four minor SNPs that were linked to haplotypes associated with heightened pain sensitivity: rs769224 (800A) was carried with the APS haplotype, while rs6267 (417T), rs740602 (422A) and rs8192488 (641T) were carried with the HPS haplotype. Subsequent *in vitro* and *in silico* experiments were performed to assess the functional impact of minor SNPs on mRNA secondary structure formation, RNA transcription, protein translation, and enzymatic activity.

## Results

### EST-based identification of minor SNPs linked to common haplotypes of the *COMT* gene

We first investigated the occurrence of SNPs within the *COMT* transcript region with lower frequency compared to commonly observed SNPs constituting the three major haplotypes. To identify minor *COMT* SNPs potentially linked to major haplotypes, we searched the existing NCBI expressed sequence tag (EST) database. As this database contains over 6 million human ESTs obtained from many unrelated sources, the variation in ESTs reflects genotypic variation naturally existing in the population.

We identified 939 human ESTs that shared greater than 95% sequence similarity over 100 nucleotides with *MB-COMT* mRNA. Of these, 306 ESTs included the positions of all three SNPs in the coding region (rs4633, rs4818 and rs4680) needed to identify the major *COMT* haplotypic structure. Based on their sequence, 138 ESTs were assigned to the LPS haplotype, 109 to the APS haplotype, and 53 to the HPS haplotype. Six ESTs were not linked to any of the three major *COMT* haplotypes and were thus excluded. Of the remaining 300 ESTs, 19 carried one known SNP with frequency of at least 1% ([41]; Table 1). Six ESTs carried the minor 417T allele of SNP rs6267, 4 carried the minor 422A allele of SNP rs740602, 3 carried the minor 641T allele of SNP rs8192488, and 6 carried the minor 800A allele of SNP rs769224. The 800A minor SNP was linked to the APS haplotype, while the 417T, 422A and 641T minor SNPs were linked to the HPS haplotype.

**Table 1:** EST-based identification of minor SNPs linked to common COMT haplotypes.

			EST	BM423612.1	BX342854.1	BX442436.2	BX381308.2	BX381309.2	BX339791.2	CA489078.1	AU133795.1	AU133795.1	BI259470.1	BF982412.1	BF982354.1	AA452360.1	BG282857.1	BF988028.1	BF988264.1	BU856727.1	CA489131.1	BU538755.1	CA489448.1	CB995968.1	CB994006.1
Position	Reference SNP	Major Allele	Minor Allele Freq																						
389	rs4633	c>t	36.33%	c	t	c	c	c	c	c	c	c	c	c	c	c	c	c	c	t	t	t	t	t	t
417	rs6267	g>t	2%	g	g	g	**t**	**t**	**t**	**t**	**t**	**t**	g	g	g	g	g	g	g	g	g	g	g	g	g
422	rs740602	g>a	1.33%	g	g	g	g	g	g	g	g	g	**a**	**a**	**a**	**a**	g	g	g	g	g	g	g	g	g
611	rs4818	c>g	46%	g	c	c	c	c	c	c	c	c	c	c	c	c	c	c	c	c	c	c	c	c	c
641	rs8192488	c>t	1%	c	c	c	c	c	c	c	c	c	c	c	c	c	**t**	**t**	**t**	c	c	c	c	c	c
675	rs4680	g>a	36.33%	g	a	g	g	g	g	g	g	g	g	g	g	g	g	g	g	a	a	a	a	a	a
800	rs769224	g>a	2%	g	g	g	g	g	g								g			**a**	**a**	**a**	**a**	**a**	**a**
Haplotype				L	A	H	H	H	H	H	H	H	H	H	H	H	H	H	H	A	A	A	A	A	A
				P	P	P	P	P	P	P	P	P	P	P	P	P	P	P	P	P	P	P	P	P	P
				S	S	S	S	S	S	S	S	S	S	S	S	S	S	S	S	S	S	S	S	S	S

Compared to SNPs constituting the LPS, APS, and HPS haplotypes, the above minor SNPs were less frequent in the population and did not coexist with one another. Therefore, these minor SNPs likely represent younger mutations that should be linked to the major haplotypes [42]. Interestingly, there was an inverse relationship between the frequency of minor SNPs and major haplotypes in the EST database. Three minor SNPs were identified in the least frequent HPS haplotype, one minor SNP was identified in the APS haplotype, and no minor SNPs were identified in the most frequent LPS haplotype. As LPS is the older haplotype, showing inter-species conservation at the level of RNA secondary structure [37], deviation from this haplotype may have functional implications- negative or positive. The polymorphic pattern exhibited by the more recent APS and HPS haplotypes could indicate the evolution of compensatory SNPs that counteract deleterious effects of low activity haplotypes [39], [43], [44]. Alternatively, the presence of additional variation in the low activity APS and HPS haplotypes could simply be neutral or nearly neutral. Thus, subsequent studies were conducted to determine if these minor frequency SNPs naturally occurring within the APS and HPS haplotypes produce compensatory, neutral, or negative effects on COMT enzymatic activity at various levels along the canonical pathway from gene to RNA to active protein.

### Effect of minor SNPs on mRNA secondary structure associated with common *COMT* haplotypes

Next, we tested whether minor SNPs linked to haplotypes associated with heightened pain sensitivity restore function of the COMT enzyme through modification of mRNA secondary structure which impacts protein translation efficiency [45]. The 800A and 641T minor alleles carried with the APS and HPS haplotypes, respectively, are situated in a stem-loop structure in the *val* ^158^ region shown to be important for COMT protein translation [37]. Thus, 800A and 641T minor alleles may modify this local stem-loop and ultimately affect protein translation. While the 417T and 422A minor alleles carried with the HPS haplotype are located further away from the *val* ^158^ region in linear space, they could interact with this region when folded or alter a remote stem-loop structure also important for protein translation.

**Figure 1:**
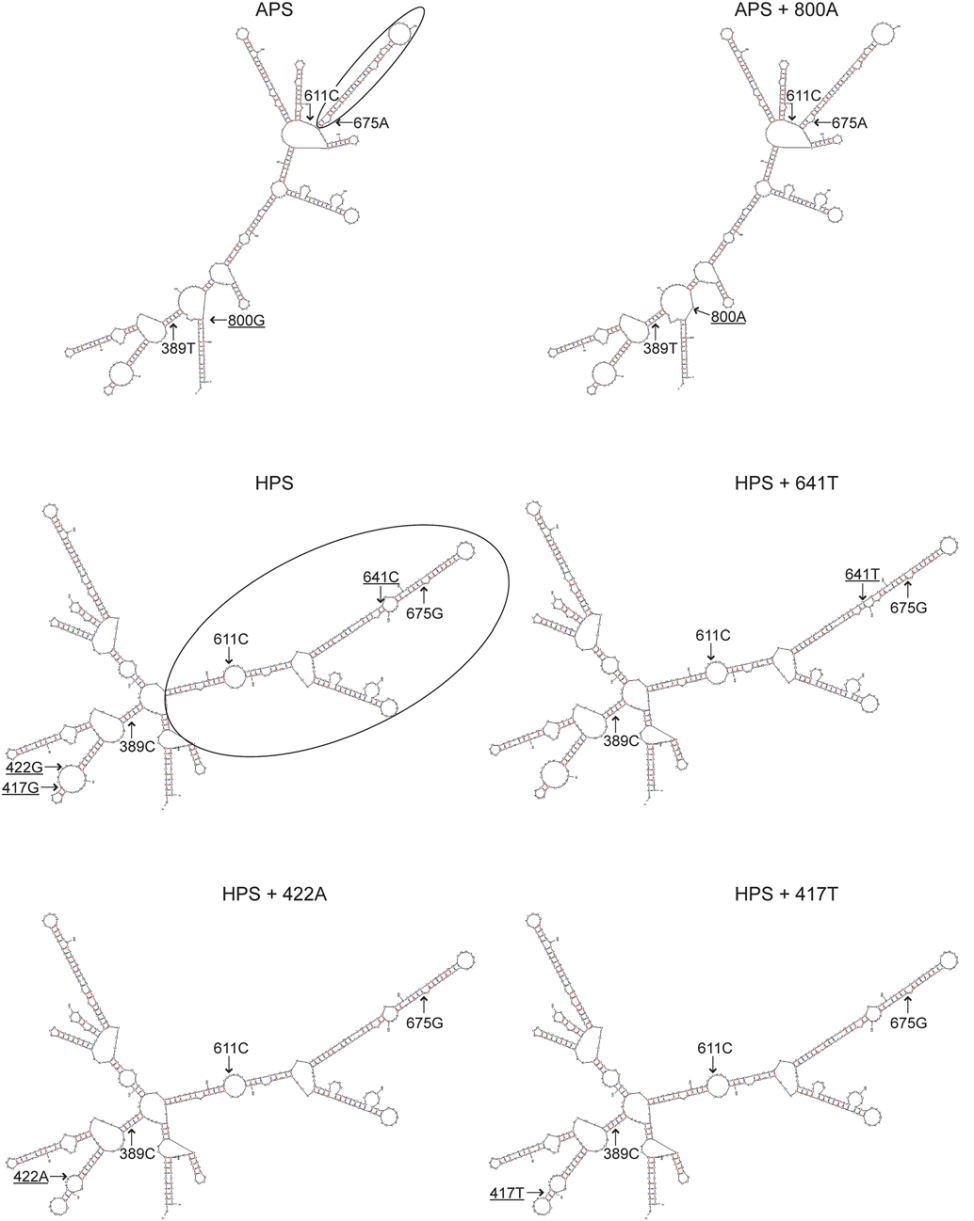
Effect of minor SNPs linked to APS and HPS haplotypes on predicted mRNA secondary structures. Polymorphic alleles C389T, C611G, and G675A that define the three major haplotypes are indicated, minor SNPs underlined, and the major functional RNA stem-loop structure associated with APS and HPS circled. Relative to the APS haplotype, the HPS haplotype coded for a longer, more stable secondary structure. Transcripts carrying the 800A or 641T mutations in the *val* ^158^ region or the 417T or 422A mutations in the nearby stem-loop did not significantly alter mRNA secondary structure.

To determine whether minor SNPs linked to APS and HPS haplotypes modify mRNA secondary structure, secondary structures were predicted for the APS, APS+800A, HPS, HPS+417T, HPS+422A, and HPS+641T mRNA transcripts using the RNA Mfold [46] and Afold [47] programs. Several structural domains were predicted within the 449 nucleotide region that contained the previously studied *val* ^158^ SNP as well as the newly studied minor SNPs (Fig. 1). In line with previous studies [37], the HPS haplotype codes for a longer more stable secondary structure in the *val* ^158^ region relative to the APS haplotype. The 800A and 641T minor alleles located within the structural domain of the *val* ^158^ region did not significantly alter this local stem-loop structure or nearby structural domains associated with the APS and HPS haplotypes, respectively. The 417T or 422A minor alleles were located outside of the structural domain of the *val* ^158^ region, within separate stem-loop structures. Inclusion of either 417T or 422A minor alleles in the HPS haplotype did not alter their local stem-loop structure or that of the nearby structural domain of the *val* ^158^ region. These computer modeling results as well as potential independent effects of minor SNPs carried with the major haplotypes were studied further in *in vitro* cell transfection experiments.

### Effect of minor SNPs on COMT RNA abundance, protein expression, and enzymatic activity

To assess the potential functional impact of minor SNPs at the level of RNA transcription, PC12 cells were transiently transfected in duplicate with expression constructs corresponding to the major LPS, APS, and HPS haplotypes as well as the APS (APS+800A) and HPS (HPS+417T, HPS+422A, and HPS+641T) haplotypes containing individual alleles of minor SNPs and then RNA abundance was measured using real time PCR.

**Figure 2:**
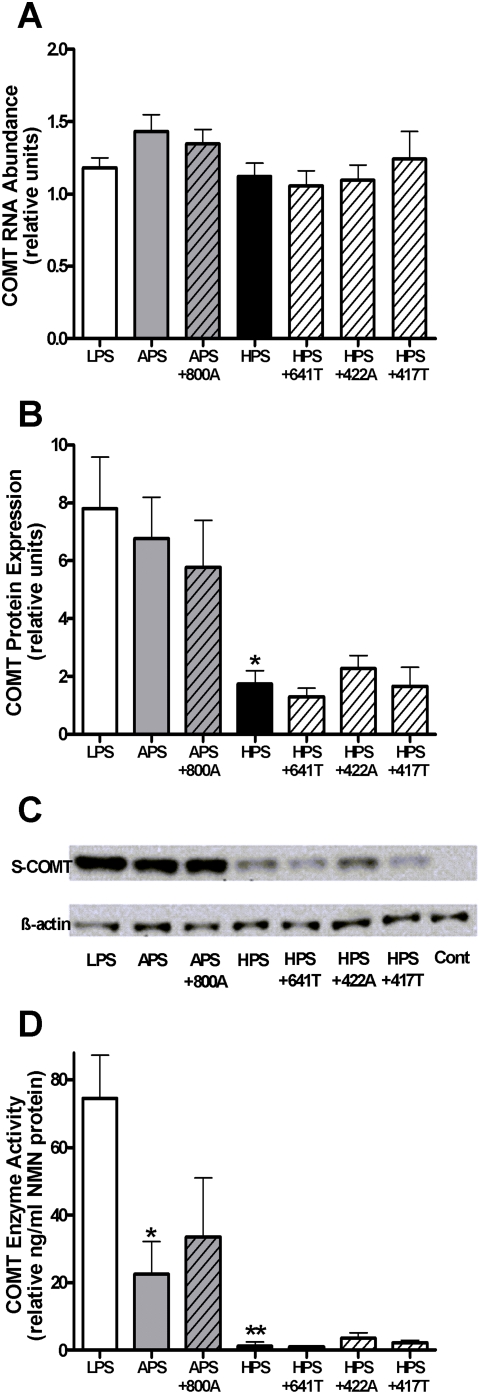
Effect of minor SNPs linked to the APS and HPS haplotypes on COMT RNA abundance, protein expression, and enzymatic activity. (A) The relative abundance of RNA was uniform among cells transfected with constructs corresponding to the LPS, APS, and HPS haplotypes. Inclusion of the 800A mutation in the APS construct or the 417T, 422A, or 641T mutation in the HPS construct did not alter RNA abundance relative to that exhibited by the respective parent haplotypes. Data from (B) independent and (C) pooled Western blot experiments reveal that in cells expressing COMT, the HPS haplotype exhibited a significant reduction in protein expression compared to the LPS haplotype. This effect was not altered by inclusion of the 417T, 422A, or 641T mutations. (D) Both the APS and HPS haplotypes showed reduced enzymatic activity compared to the LPS haplotype. The modest reduction in enzymatic activity displayed by the APS haplotype and the marked reduction displayed by the HPS haplotype was not altered by inclusion of individual allelic variants of minor SNPs. Data are Mean±SEM. *P<0.05 and **P<0.01 different from the LPS haplotype.

In agreement with results from previous reports [27], [37], LPS, APS, and HPS haplotypes exhibited uniform levels of *COMT* RNA abundance (Fig. 2A). Inclusion of the 800A minor allele in the APS haplotype or the 417T, 422A, or 641T minor alleles in the HPS haplotype did not alter RNA abundance relative to that exhibited by the respective parent haplotypes. As previous studies demonstrated that *COMT* haplotype-dependent RNA abundance does not parallel protein expression or enzyme activity [27], [37], minor SNPs may produce effects downstream of RNA transcription.

To directly assess the potential functional impact of minor SNPs at the level of protein translation and activity, PC12 cells were transiently transfected in duplicate with constructs corresponding to the three major haplotypes or with the APS or HPS haplotype constructs containing individual allelic variants of minor SNPs (as listed above). Protein expression and enzymatic activity were subsequently measured using Western blot analysis and normetanephrine ELISA, respectively.

Consistent with previous studies [37], protein expression levels exhibited by the APS haplotype did not differ relative to the LPS haplotype, while expression levels displayed by the HPS haplotype were reduced 4.5-fold (F_6,7_ = 6.13, *P*<0.02; Fig. 2B and 2C). Inclusion of the 800A minor allele in the APS haplotype or the 417T, 422A, or 641T minor allele in the HPS haplotype did not alter protein expression relative to that exhibited by the respective parent haplotypes.

Also consistent with previous studies [37], enzymatic activity corresponding to both the APS and HPS haplotypes was reduced compared to the LPS haplotype (F_6,7_ = 9.24, *P*<0.005; Fig. 2D). The APS haplotype showed a moderate 3.5-fold reduction, while the HPS haplotype displayed a marked 58-fold reduction in enzymatic activity. Again, inclusion of individual minor alleles in the APS or HPS haplotypes did not produce effects significantly different from those associated with the parent haplotypes. It is important to note that the NMN ELISA has adequate sensitivity to detect reductions in enzymatic activity beyond those associated with the HPS haplotype. Cells transfected with clones corresponding to the HPS haplotype exhibited 5.6 ng/ml absolute NMN protein (the enzymatic activity values displayed in Figure 2 are relative values, as defined by the y-axis label). The kit is sensitive enough to detect 0.6 ng/ml NMN protein, and thus capable of capturing a 9.3–fold decrease in enzymatic activity relative to the HPS haplotype. Taken together, these results suggest that the synonymous and nonsynonymous minor SNPs tested herein do not significantly alter functional effects on COMT enzymatic activity produced by the *met^158^* allele associated with the APS haplotype and the mRNA secondary structure associated with the HPS haplotype and, thus, can be considered neutral.

## Discussion

Our results are in line with those from previous studies demonstrating that the APS and HPS haplotypes, albeit to a different degree and through different mechanisms, both impair COMT function. The *met* ^158^ allele included in the APS haplotype codes for a ∼3-fold reduction in COMT activity, but not amount, that is due to reduced stability of the enzyme at normal physiologic temperature. The HPS haplotype codes for a ≥20-fold reduction in COMT activity that is due to the longer more stable local stem-loop structure in the *val* ^158^ region that reduces protein translation efficiency.

As an extension of this work, we next tested whether additional SNPs situated in the transcript region that occur at lower frequency in the population contribute to variation in COMT activity. We identified a total of four such minor SNPs that were linked to APS or HPS haplotypes. Interestingly, there was an inverse relationship between the frequency of minor SNPs and major haplotypes in the EST database. Three out of the four minor SNPs were linked to the least frequent HPS haplotype coding for the least COMT activity, while one was linked to the APS haplotype coding for average COMT activity, and none were linked to the most common LPS haplotype coding for the highest COMT activity. The distribution of minor SNPs exclusively within haplotypes coding for reduced COMT activity may suggest different evolutionary models (e.g., enrichment for functional compensatory mutations or positive selection leading to carrying of neutral mutations) depending on the functional contribution of these SNPs.

Compensatory SNPs have been shown to restore fitness by suppressing the deleterious effects of nonsynonymous mutations on receptor folding, DNA-binding, and enzymatic activity [31], [43], [48], [49]. Thus, minor SNPs linked to the APS haplotype may compensate for the decreased enzyme thermostability produced by the *met* ^158^ allele. Compensatory SNPs have also been shown to normalize RNA secondary structure formation altered by synonymous pathogenic mutations [38], [45], [50], [51]. Thus, minor SNPs linked to the HPS haplotype may compensate for the loss of Gibbs free energy in the RNA stem-loop structure. In contrast to compensatory SNPs, the existence of neutral SNPs in APS and/or HPS haplotypes would indicate positive selection for haplotypes associated with reduced COMT activity. As COMT regulates neuronal and non-neuronal cellular pathways important for a vast number of basic and complex biologic functions, reduced COMT leading to elevated catecholamine levels could also be beneficial.

To test whether minor SNPs linked to APS or HPS haplotypes rescue COMT activity, further reduce it, or produce neutral effects, a series of molecular modeling and cell culture studies were performed. Inclusion of the 800A (rs769224) minor allele in the APS haplotype or the 417T (rs6267), 422A (740602), or 641T (rs8192488) minor alleles in the HPS haplotype did not alter mRNA folding, RNA transcription, protein translation, or enzymatic activity. The possibility remains that inclusion of minor SNPs with APS and HPS haplotypes produces very subtle effects that were not detected using our methods and might still have effects on individual fitness or that inclusion of minor SNPs produces functional effects on molecular phenotypes not evaluated in the present study.

Therefore, these minor SNPs are likely neutral variations versus functional contributors to COMT activity. However, these data are inconsistent with a simple model of an ancestral selectively advantageous haplotype and two derived deleterious haplotypes. Under a typical population genetic model the ancestral LPS haplotype should have the highest number of neutral variants, even if undergoing strong purifying selection for high activity [52]. Likewise, the more recent APS and HPS haplotypes should have a smaller number of neutral variants if undergoing background selection for deleterious low activity [52]. Why these haplotypes are more variable than the LPS haplotype is puzzling. What evolutionary processes could cause a departure from a simple model of ancestral selectivity? One possibility is that APS and HPS haplotypes are not unconditionally deleterious, but under balancing selection due to pleiotropic effects on traits other than pain sensitivity. For example, the counterintuitive selection for low activity COMT haplotypes associated with high pain, anxiety, and stress response may be driven by a gain of cognitive function related to higher dopamine and norepinephrine levels. An inverted U-shaped relationship exists between catecholamine levels and cortical function, such that intermediate levels are optimal for cognition [3]. Reduced COMT activity would produce a rightward shift in the inverted U-shaped curve, leading to higher levels of catecholamines associated with increased cognitive function advantageous for memory and attention tasks. Thus, both high and low activity haplotypes, each with opposing advantages and disadvantages, may be maintained in the population due to balancing selection [53]. While there is no evidence, to date, of greater sequence variation in the COMT locus than that expected under a neutral model [54], the distribution of minor SNPs exclusively within haplotypes associated with reduced COMT activity requires further study. The balancing selection hypothesis would need to be addressed by 1) thorough re-sequencing of the *COMT* gene locus followed by haplotype-based and linkage-disequilibrium-based analyses [52], [55], 2) thorough phenotyping of individuals for multiple traits (e.g., pain, cognition, and memory function), and 3) development of more complex population genetic models to account for pleiotropic selection. Whether advantageous or disadvantageous phenotypes associated with low activity COMT haplotypes are ultimately expressed, is probably determined by additional genetic and non-genetic factors.Although the significant functional impact of common APS and HPS haplotypes is independent of minor SNPs situated in the coding region of the gene, it is possible that effects of APS and HPS haplotypes are modified by epistatic interactions occurring at nearby *COMT* gene loci not investigated here. A previous report demonstrated that the minor allele of rs2097603 located in the *MB-COMT* promoter region associated with schizophrenia [34] was found to produce a 1.5-fold reduction in lymphocyte COMT activity independent of the val^158^met allele [56]. Additionally, a haplotype consisting of two noncoding SNPs, rs737865 in intron 1 and rs165599 in the 3′ untranslated region was associated with Schizophrenia [35] and reduced expression of *COMT* mRNA [57]. These SNPs are not in high LD with the SNPs constituting the three major haplotypes, however may still interact with the coding SNPs to influence the net size and direction of effect. Furthermore, a haplotype consisting of the minor alleles of rs737865 and rs4818 in the HPS haplotype are associated with increased thermal threshold variance, implicating a role for additional unobserved functional polymorphisms [40]. It is also possible that the effects of APS and HPS haplotypes are modified by epistatic interactions with mutations located in convergent molecular pathways. For example, hyperhomocysteinemia is an important risk factor for a variety of conditions associated with low COMT activity, including neurodegenerative disorders, cardiovascular disease, and hormonal cancers [58]. Homocysteine is a precursor for the biosynthesis of S-adenosyl-L-homocysteine (SAH) which is a strong, noncompetitive inhibitor of COMT. Functional polymorphisms in genes relevant to homocysteine metabolism (e.g., 5,10-methylenetetrahydrofolate reductase; MTHFR and glutamate carboxypepidase II; GCPII [59]–[61]) that result in accumulation of SAH likely augment the effects of APS and HPS haplotypes. In fact, a recent report demonstrated that individuals homozygous for low enzyme activity alleles of the *COMT* and *MTHFR* genes in combination, but not alone, are at increased risk for schizophrenia [62]. Thus, it is possible that minor SNPs situated within APS and HPS haplotypes compensate for low COMT activity not at the level we define, but instead through interactions with other genes or genotypes.

Additionally, the effects of APS and HPS haplotypes may be modified by nongenetic factors, such as environmental events that lead to a sustained elevation in catecholamines (e.g., physical and emotional stress, inflammation, and injury [63]–[65]) and nutritional deficiencies that contribute to hyperhomocysteinemia (e.g., reduced dietary folate, vitamin B_12_, or vitamin B_6_ [66], [67]). An example of *COMT* gene-environment interaction was recently illustrated by Slade and colleagues, who found that among individuals with the APS or HPS haplotype, TMD incidence was 23% for those with a history of orthodontic treatment and 0% for those with no history of orthodontic treatment [68]. In order to truly understand the dynamic etiology of complex catechol-dependent phenotypes, flashlights used to examine the relationship between one SNP and one phenotype need to be traded in for floodlights that will illuminate dynamic relationships between genetic and epigenetic factors. Thus, future studies necessitate careful consideration as to how polymorphisms within and between distinct genetic loci interact with one another as well as with nongenetic factors to place individuals at risk.

The 417T allele was previously associated with clinical effects. This minor SNP, producing an alanine to serine substitution at codon 72, has been associated with aggressive behavior and schizophrenia risk as well as with COMT activity in red blood cells collected from study participants [32], [33]. However, we found that the functional impact of common APS and HPS haplotypes is independent of this less frequent second-site mutation situated in the coding region of the human *COMT* gene. In light of this, we can revisit the conclusion regarding effects previously associated with 417T. In that study, individuals homozygous for 417G (*ala* ^72^) had the highest activity, while those homozygous for 417T (*ser* ^72^) had the lowest activity. Individuals exhibiting both the highest and lowest COMT activity were homozygous for the *val* ^158^ allele, which is present in both the LPS and HPS haplotypes. In correspondence with the APS haplotype, individuals exhibiting average COMT activity were homozygous for the *met* ^158^ allele. As 417T is carried exclusively with the HPS haplotype and its inclusion in this parent haplotype does not alter enzymatic activity, we can conclude that 417T is a marker of HPS haplotype and that the low activity associated with the nonsynonymous *ser* ^72^ allele is in fact due to alterations in RNA secondary structure and protein translation efficiency coded for by the parent HPS haplotype.

In summary, we demonstrated that the impact of common *COMT* haplotypes on enzymatic activity is independent of additional minor SNPs in the coding region of the human *COMT* gene. Tight linkage of these neutral polymorphisms with more recently acquired low activity APS and HPS haplotypes does not satisfy a simple ancestral model and requires the development of a more complex population genetic model to account for pleiotropic selection. The counterintuitive selection for low activity *COMT* haplotypes may be driven by a gain of cognitive function, for example, related to increased catecholamine levels. Thus, the expression “no pain – no gain” may have literal meaning when applied to evolution of the human *COMT* locus.

## Materials and Methods

### EST database analysis

Using an approach similar to that used previously [69], we first performed a BLAST search of the human EST database (dbEST release May 09, 2008) to identify all allelic combinations occurring within the complete nucleotide sequence of *MB-COMT* mRNA (NM_000754.2, length = 1289 nucleotides). We then selected all matching ESTs longer than 100 nucleotides, so that at least 80% of their length overlapped with the COMT sequence with 95% similarity. The list of ESTs was then constrained to those containing all three SNPs in the coding region (rs4633, rs4818 and rs4680) needed to identify the three major *COMT* haplotypes. Analysis of nucleotide variation within the three haplotypes was restricted to SNPs with frequency of at least 1%. The minor allele frequency of minor SNPs in our constrained EST list corresponded to that annotated in the NCBI dbSNP.

### Prediction of RNA secondary structure

Secondary structures of the full-length APS, APS+800A, HPS, HPS+417T, HPS+422A, and HPS+641T mRNA transcripts were predicted using the RNA Mfold program (versions 3.1 and 3.2) [46] and the Afold program [47]. Energy minimization was performed by a dynamic programming method that finds the secondary structure with the minimum free energy with sums comprised of stacking, loop length, etc. [47], [70]. The RNA folding parameters were developed and published by the Turner group [71]. Suboptimal stem-loop structures were analyzed by the Hybrid program [72], [73] for the full-length *COMT* transcripts, and for truncated transcript sequences of different lengths ranging from the rs4633 to the rs769224 region with 449 nucleotide window length.

### Construction of COMT variants

Previously constructed full-length cDNA COMT clones that differed only in three nucleotides corresponding to the LPS, APS, and HPS haplotypes were used [37]. Four individual minor SNPs were introduced in the parent APS (rs769224; 800G→A) or HPS (rs6267; 417G→T, rs740602; 422G→A, or rs8192488; 641C→T) constructs by site directed mutagenesis using the Quickchange II XL Site-Directed Mutagenesis Kit (Stratagene, La Jolla, CA, USA). Plasmid DNA was purified using the EndoFree Plasmid Maxi purification kit (Qiagen, Germantown, MD, USA). Once plasmids were isolated, DNA sequences were confirmed by double sequencing at the UNC core sequencing facility.

### Transient transfection of COMT cDNA clones

A rat adrenal cell line (PC-12) was transiently transfected in six-well plates using FuGENE 6 Transfection Reagent (Roche, Basel, Switzerland) in accordance with manufacture's recommendations. The amount of plasmid was kept at 1 µg/well. Cells were cotransfected with pSV-βGalactosidase (0.1 µg/well) and SEAP (0.1 µg/well) plasmids to control for transfection efficiency and RNA abundance, respectively (Promega, Madison, WI, USA). Transfections with the vector lacking the insert were also done for each experiment. The same cell line was used for analysis of total RNA, protein expression, and enzymatic activity. Transfection experiments for analysis of total RNA and protein expression/enzymatic activity were performed in duplicate concurrently. Cell lysate was then collected approximately 48 hours post-transfection.

### Real-time PCR

Total RNA was isolated using the Trizol reagent (Invitrogen, Carlsbad, CA, USA). The isolated RNA was treated with RNase free-DNase I (Promega) and reverse transcribed by Superscript III reverse transcriptase (Invitrogen). The cDNA for COMT and SEAP was amplified with SYBR Green PCR Master Mix (Applied Biosystems, Foster City, CA, USA) using forward and reverse PCR primers (TGAACGTGGGCGACAAGAAAGGCAAGAT and TGACCTTGTCCTTCACGCCAGCGAAAT, respectively, for COMT and GCCGACCACTCCCACGTCTT and CCCGCTCTCGCTCTCGGTAA, respectively, for SEAP). SEAP was used to normalize *COMT* RNA abundance for transfection efficiency. The MCEP Realplex 2S System (Eppendorf, Westbury, NY, USA) was used for measuring fluorescence.

### Western blot

Purified lysates, normalized for protein content using a BCA assay, were run on 12% Novex Tris-Glycine gels (Invitrogen) and transferred to nitrocellulose membranes (Whatman, Florham Park, NJ, USA). Blots containing COMT protein were blocked with 5% nonfat milk for 30 min at room temperature, incubated with COMT polyclonal primary antibody (1∶10,000; Chemicon, Temecula, CA, USA) overnight at 4°C, and then incubated with Goat Anti-Rabbit IgG HRP polyclonal secondary antibody (1∶10,000; Chemicon) for 1 hr at room temperature. Blots were washed with PBST for 10 min at RT, exposed to chemiluminescence reagent (Pierce, Milwaukee, WI, USA), and developed. Blots were then stripped using Restore western stripping buffer (Pierce) and equal loading of samples verified by β-actin staining. Blots were incubated with β-actin polyclonal primary antibody (1∶10,000; Santa Cruz Biotechnology, Santa Cruz, CA, USA) for 1 hr at RT followed by Goat Anti-Rabbit IgG HRP polyclonal secondary antibody (1∶10,000; Chemicon) for 1 hr at RT and chemiluminescent reagent.

### Enzymatic assay

After removal of the media, the cells were washed twice with 0.9% saline (1 ml/well) and covered with deionized water containing 10 mM CDTA (300 µl/well). The cells were freeze/thawed (−80°C/room temperature) five times and the lysate collected in 1.7 ml tubes. The tubes were centrifuged at 2,000 g for 20 minutes and the filtrate removed. The enzymatic COMT assay procedure followed the protocol described by Masuda's group [74]. Purified lysates (8 µl) were incubated with 200 µM Sadenosyl-L-methionine (SAMe; ICN Chemicals, Aurora OH, USA), 7.5 mM L-norepinephrine (NE; Sigma Chemical Co., St. Louis MO, USA) and 2 mM MgCl2 in 50 mM phosphate buffered saline for 60 min in the final volume of 22 µl. The reaction was terminated using 20 µl of 0.4 M hydrocholoric acid and 1 µl of 330 mM EDTA. The same reaction in the presence of 15 mM EDTA was carried out in parallel for each lysate to bind Mg^+2^ ions required for COMT activity. COMT activity was assessed as measurement of normetanephrine (NMN) by Normetanephrine ELISA kit (IBL, Hamburg, Germany) in accordance with manufacture's recommendations using 10 µl of above reaction mixture. COMT activity was determined after subtracting the amount of NMN produced by endogenous enzymatic activity (transfection with empty vector). The non-specific background was determined in parallel assays performed in the presence of EDTA and then subtracted from each reading. COMT activity was then normalized for transfection efficiency by measuring the β-galactosidase activity for each lysate. β-galactosidase activity was determined using β-galactosidase enzyme systems (Promega) in accordance with manufacture's recommendations. The standard curve for ELISA was determined using a 4 parameter sigmoidal dose-response model for one-site competitive binding systems. The lowest detectable level that can be distinguished from the zero standard is 0.6 ng/ml.

### Statistical analysis

COMT RNA abundance, protein expression, and enzyme activity levels from duplicate experiments were analyzed by one-way analysis of variance (ANOVA). Post hoc comparisons were performed using Bonferroni's Multiple Comparison Test. P<0.05 was considered significant.
